# Exploring the Diversity of Active Ureolytic Bacteria in the Rumen by Comparison of cDNA and gDNA

**DOI:** 10.3390/ani10112162

**Published:** 2020-11-20

**Authors:** Sijia Liu, Nan Zheng, Shengguo Zhao, Jiaqi Wang

**Affiliations:** State Key Laboratory of Animal Nutrition, Institute of Animal Sciences, Chinese Academy of Agricultural Sciences, No.2 Yuanmingyuan West Road, Haidian, Beijing 100193, China; liusijia1214@163.com (S.L.); zhengnan@caas.cn (N.Z.); jiaqiwang@vip.163.com (J.W.)

**Keywords:** rumen, ureolytic bacteria, active, diversity

## Abstract

**Simple Summary:**

Ureolytic bacteria produce urease that hydrolyzes dietary or recycled urea to ammonia, which can then be converted into microbial proteins. The diversity of ruminal ureolytic bacteria benefits N utilization efficiency in ruminants. However, there is no information at the complementary DNA (cDNA) level to reflect the active status of ureolytic bacteria. To reveal the diversity of active ureolytic bacteria in the rumen, we compared *ureC* amplicons between genomic DNA (gDNA) and cDNA. These results revealed distinct ureolytic bacterial community profiles based on gDNA and cDNA. The dominant ureolytic bacterial had high transcriptional activity, and the differential were mainly distributed in the genus of low abundance.

**Abstract:**

In this study we revealed the diversity of active ureolytic bacteria in the rumen by compared *ureC* amplicons between gDNA and cDNA. Rumen fluid was collected from four Holstein dairy cows with rumen fistulas at 0, 2, and 6 h after morning feeding. Total microbial gDNA and RNA were isolated, and the RNA was reverse-transcribed into cDNA. The *ureC* gene amplicons of gDNA and cDNA were produced and sequenced by MiSeq. These results revealed that the sampling time had no significant difference on the alphssa and beta diversity indices of the ureolytic bacteria. The Shannon diversity of the *ureC* gene for cDNA was greater than that for gDNA (*p* < 0.05). There were significant difference in the beta diversity of ureolytic bacteria between gDNA and cDNA (*p* < 0.01), which indicates a shift in the community of active ureolytic bacteria. Approximately 67% of *ureC* sequences from cDNA could not be confidently classified at the genus level. The active ureolytic bacteria were mainly from *Helicobacter*, *Herbaspirillum*, *Clostridium*, *Paenibacillus*, *Synechococcus*, and *Sphingobacterium* sp. Changes in the operational taxonomic units revealed that the top abundant *ureC* genes were mostly consistent between gDNA and cDNA, and most differences occurred in the *ureC* genes with lower abundances. These results revealed distinct ureolytic bacteria community profiles based on gDNA and cDNA. The dominant ureolytic bacteria had high transcriptional activity, and the differential were mainly distributed in the genus of low abundance.

## 1. Introduction

Urea is an economical non-protein nitrogen used in the diets of ruminants [1]. Ureolytic bacteria produce ureases in the rumen, which is a key enzyme in the hydrolysis of dietary urea to ammonia and carbon dioxide [2]. Ammonia derived from urea can conversion into microbial proteins, which are ideal to promote animal growth and production [3]. Endogenous urea is converted in the liver from excess ammonia and then recycled via the ruminal wall and salivary secretion [4]. Urea recycling is an important biological process in the rumen in response to low dietary nitrogen [5]. However, hyperactivity of urease activity produces excess ammonia that is excreted in the urine, which reduces the quality of lactoprotein and pollutes the environment. Therefore, elucidation of diversity of rumen ureolytic bacteria is important to increase the efficiency of urea-N utilization for ruminants.

We assessed the diversity of ureolytic bacteria in the rumen, based on analysis of gDNA [6]. Although gDNA-based techniques have provided important insights, there are persistent shortcomings in the ability to identify active microbes in the rumen [7]. The detected gDNA may originate from dead or inactive bacteria [8]. A large number of *ureC* genes have been identified in the rumen, but the expression patterns of these genes remain unclear. In general, RNA is reportedly a more reliable indicator of bacteria viability than target genes of gDNA [9,10,11]. Qi et al. [12] examined fungal gene expression in the rumen of muskoxen (*Ovibos moschatus*) by analysis of a library of cDNA.

Sequencing of cDNA amplicons from reverse-transcription of RNA is better suited to assess the in situ activity of the microbial community because the concentration of RNA is generally related to the synthesis potential and activity of microbial proteins [13]. RNA levels are Under stress, endonuclease can initiate degradation of functional ribosomes, whereas homologs in physically damaged or dying bacteria can be degraded by homologs of ribonuclease I [14]. The bacteria ribosomes relatively labile characteristic has been used in numerous studies to better assess the active and viable components of microbial communities [14,15].

To investigate the diversity of active ureolytic bacteria, we compared *ureC* amplicons between gDNA and cDNA in the rumen. This survey is expected to expanded current knowledge of the active ureolytic microbial community in the rumen in order to provide a basis for the design of regulation rumen ureolytic bacteria composition to increase the efficiency of urea-N utilization for ruminants.

## 2. Materials and Methods

### 2.1. Animals and Sampling

Rumen fluid samples were collected from four non-lactating Holstein dairy cows (body weight, 550 ± 50 kg) fitted with ruminal fistulas. The experimental procedures involving the care and management of dairy cows were approved by the Animal Care and Use Committee for Livestock of the Institute of Animal Sciences, Chinese Academy of Agricultural Sciences (No. IAS201914). Diet was a total mixed ration, consisting of 36% corn silage, 16% corn, 14.6% syrup vinasse, 5.9% soybean meal, 5.7% dried distillers grains with solubles, 5.4% soybean hulls, 5.3% barley, 5.0% oat grass, 4.0% alfalfa, 0.8%, CaHPO_4_, 0.5% NaHCO_3_, 0.2% NaCl, 0.2% CaCO_3_, 0.1%, C_5_H_14_ClNO, 0.1% calcium fatty acid, 0.1% double beneficial element, and 0.1% rhodamine. Samples of the rumen contents of each cow were obtained at 0, 2, and 6 h after morning feeding. The rumen fluid samples were stored in liquid nitrogen prior to analysis.

### 2.2. gDNA Extraction and cDNA Reverse Transcription

Total gDNA was extracted using the cetyltrimethylammonium bromide method, as described previously [16]. Total RNA was extracted using TRIzol method. Rumen fluid samples were homogenized for 5 min with steel balls (one with a diameter of 20 mm and 10 with diameters of 5 mm) into fine powder in liquid nitrogen using a CryoMill (Retsch GmbH). Then, 3 g of the rumen fluid samples were incubated in 15 mL of TRIzol reagent (Invitrogen Corporation, Carlsbad, CA, USA) at room temperature for 5 min. Afterward, 4 mL of chloroform were added to the samples by vortexing for 15 s and then each was incubated at room temperature for 3 min. Following centrifugation at 13,000× *g* for 15 min at 4 °C, the aqueous phase was transferred to a fresh tube. Following the addition of 10 mL of isopropyl alcohol, the tubes were vortexed for 30 s and then incubated at room temperature for 10 min. Afterward, the samples were centrifuged at 13,000× *g* for 10 min at 4 °C and the supernatant was discarded. The RNA pellet was washed once with 75% ethanol and resuspended in 10 mL of 75% ethanol by hand mixing and then centrifuged at 7500× *g* for 10 min at 4 °C. Once the supernatant was removed, the RNA pellet was air-dried and dissolved in RNase-free water by passing the solution a few times through a pipette tip, and then incubated for 10 min at 55 °C. Trace gDNA in the RNA samples was removed by incubation with 4 μL of DNase I (Takara Bio, Inc., Kusatsu, Shiga Prefecture, Japan) per 100 μg of total RNA for 30 min at 37 °C. Then, the RNA was further purified using an RNAclean Kit (Tiangen Biotech Co., Ltd., Beijing, China). The 16S rRNA gene was amplified using the primers 27F (5′-GAG TTT GAT CCT GGC TCA G-3′) and 1492R (5′-GGT TAC CTT GTT ACG ACT T-3′). Purified RNA was used to check whether the purified RNA samples contained residual gDNA. The integrity and concentration of the total RNA were assessed using an Agilent 2100 Bioanalyzer (Agilent Technologies, Mississauga, ON, Canada). RNA samples with an RNA integrity number > 8.0 were deemed suitable for reverse transcription of cDNA, which was synthesized using FastQuant RT Super Mix (Tiangen Biotech Co., Ltd.) and random primers in accordance with established procedures. 

### 2.3. PCR Amplification and Sequencing of UreC Genes

*UreC* genes were amplified with the primer set *UreC*-F (5′-TGG GCC TTA AAA THC AYG ARG AYT GGG-3′) and *UreC*-R (5′-GGT GGT GGC ACA CCA TNA NCA TRTC-3′) [17]. Reactions were performed in a MyCycler Thermal Cycler (Bio-Rad, Hercules, CA, USA) using a 50-µL mixture containing 5 µL of 10 × PCR buffer (Invitrogen Corporation), 1 µL of dNTP mixture (10 mM), 1.5 µL of each forward and reverse primer (10 µM), 0.25 µL of Platinum Taq DNA polymerase (Invitrogen Corporation, Carlsbad, CA, USA), 2 µL of gDNA or cDNA (100 ng/µL), and 38.75 µL of sterile double-distilled H_2_O. The PCR amplification consisted of denaturation at 94 °C for 5 min, followed by 30 cycles at 94 °C for 30 s, 50 °C for 30 s, and 72 °C for 30 s, and a final extension at 72 °C for 15 min [6]. PCR amplicons were extracted from agarose gels and purified using the AxyPrep DNA Gel Extraction Kit (Axygen Scientific Inc., Union City, CA, USA) in accordance with the manufacturer’s instructions and quantified using the QubitTM Assay kit (Thermo Fisher Scientific, Waltham, MA, USA). Purified amplicons were pooled in equimolar amounts and paired ends were sequenced (2 × 300 bp) on an Illumina MiSeq platform (Illumina, Inc., San Diego, CA, USA) by Shanghai Majorbio Bio-pharm Technology Co., Ltd. (Shanghai, China). All the sequences were submitted to the NCBI Sequence Read Archive (SRA; http://www.ncbi.nlm.nih.gov/Traces/sra/), under accession number SRA: SRP278349.

### 2.4. Statistic Analysis

Sequence analysis used methods described previously [6]. Briefly, low-quality raw reads were eliminated using the Trimmomatic read trimming tool [18]. Paired-end reads were merged using FLASH [19]. Merged reads of >200 bp were assigned to each sample based on the unique barcode [20,21]. Chimera sequences were detected and removed using the UCHIME de novo algorithm [22]. Operational taxonomic units (OTUs) were clustered at a similarity cut off value of 0.97 using the USEARCH sequence analysis tool included with the QIIME package [20,23]. A clustering value of 0.97 similarity was empirically confirmed by analyzing the clustering of taxonomically known *ureC* genes [6]. Taxonomic assignment of representative sequences of OTUs was performed using GraftM 1 with a likelihood cut off of 0. 80 when using pplacer for placement of the sequences against a previously compiled *ureC* gene package [6]. Alpha and beta diversity indices and significant fold-changes of OTUs were calculated with QIIME. 

The differences between gDNA and cDNA for the relative abundance > 0.001% and 1% *ureC* gene OTUs and ureolytic bacteria genera, respectively, were analyzed using the paired Mann–Whitney test with GraphPad Prism software (GraphPad Software, Inc., San Diego, CA, USA). Differences in beta diversity between gDNA and cDNA were determined by analysis of similarity. A probability (*p*) value of < 0.05 was considered statistically significant.

## 3. Results

### 3.1. Alpha Diversity of UreC Genes

In total, 321,612 quality sequence reads were obtained. The average number of reads was 14,619. The total sequences were assigned to 676 OTUs at a sequence similarity cut-off of 97%.

**Figure 1:**
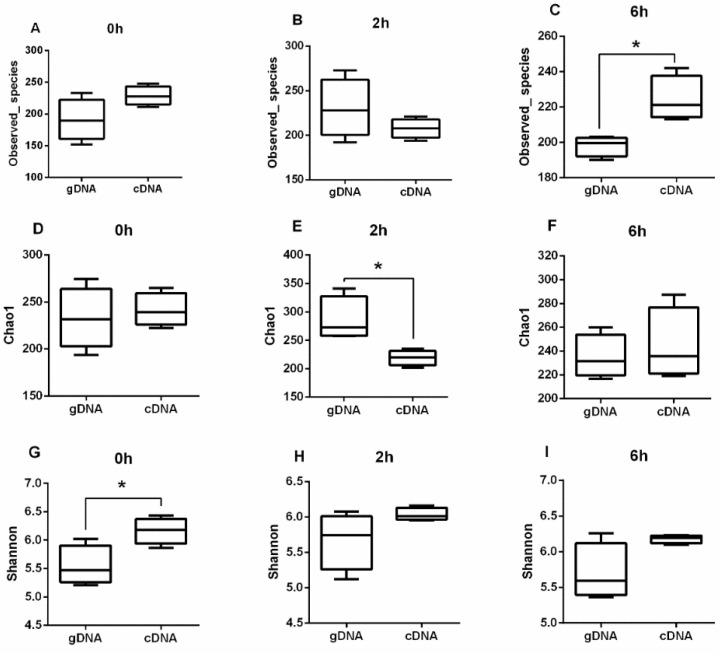
Alpha diversity of rumen ureolytic bacteria across gDNA and cDNA at different time. (**A**) Total observed species, (**B**) Chao1, and (**C**) Shannon index on at 0 h. (**D**) Total observed species, (**E**) Chao1, and (**F**) Shannon index on at 2 h. (**G**) Total observed species (**H**) Chao1 and, (**I**) Shannon index at 6 h. Boxplots indicate the first and third quartiles with the median value indicated as a horizontal line the whickers extend to 1.5 times the inter quartile range. * *p* < 0.05.

Based on the *ureC* genes derived from gDNA and cDNA samples from different time points, there were no significant differences in the Observed_species, Chao1, and Shannon diversity indices (Figure S1) (*p* > 0.05). The results indicated that the sampling time had no significant difference on the alpha diversity of gDNA and cDNA. The cDNA had higher Shannon diversity indices at 0 h, while the Chao1 indices significantly decreased after 2 h and the observed species significantly increased after 6 h (Figure 1) (*p* < 0.05). 

**Figure 2:**
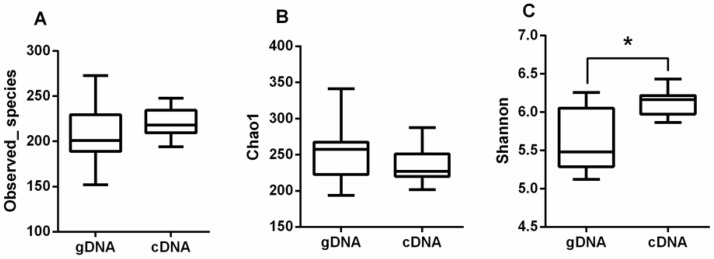
Alpha diversity of rumen ureolytic bacteria of gDNA and cDNA. (**A**) Total observed species, (**B**) Chao1, and (**C**) Shannon index. Boxplots indicate the first and third quartiles with the median value indicated as a horizontal line the whickers extend to 1.5 times the inter quartile range. * *p* < 0.05.

There were no significant differences (*p* > 0.05) in Observed_species (Figure 2A) and Chao1 (Figure 2B) for the gDNA and cDNA for all the samples from different sampling times. The Shannon index (Figure 2C) of the cDNA was greater than that of the gDNA (*p* < 0.05). The *ureC* alpha diversity of the cDNA was significantly higher than that of the gDNA.

### 3.2. Beta Diversity of UreC Genes

**Figure 3:**
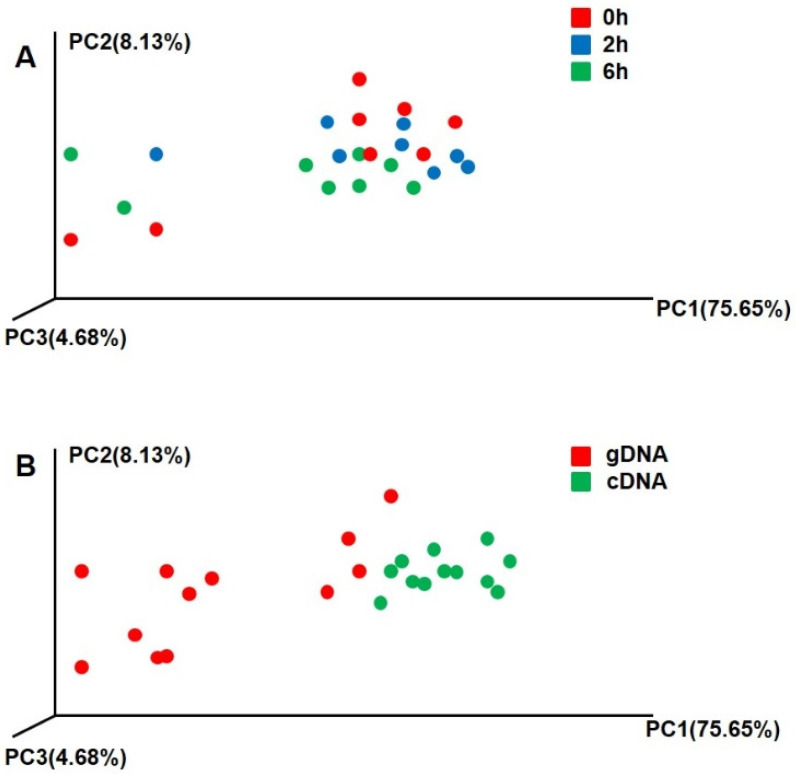
Beta diversity of rumen ureolytic bacteria of gDNA and cDNA. (**A**) Different sampling time, red dot represent 0 h, blue dot represent 2 h, green dot represent 6 h. (**B**) Ureolytic bacteria of gDNA and cDNA, red represent gDNA; green represent cDNA. Principle coordinate analysis (PCA) comparing changes in ureolytic bacteria based on weighted Unifrac distances.

The sampling time had no significant difference on the beta diversity of gDNA and cDNA (*p* > 0.05) (Figure 3A). However, there were significant differences in the beta diversities of gDNA and cDNA for all samples at different sampling times (*p* < 0.01) (Figure 3B). There were also significant differences in the compositions of active and total ureolytic bacteria.

### 3.3. Composition of Ureolytic Bacteria

**Figure 4:**
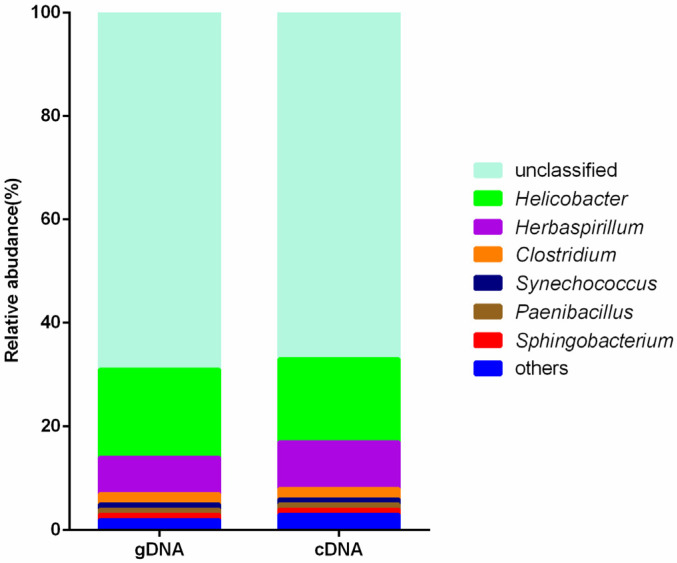
Taxonomy of the ureolytic bacteria components of gDNA and cDNA. The top 7 genera based on relative abundance are presented, the remaining genera grouped as “others”.

Approximately 67% and 69% of the cDNA and gDNA sequences, respectively, could not be confidently classified at the genus level, while the remaining sequences were assigned to *Helicobacter* (16% and 17%), *Herbaspirillum* (7% and 9%), *Clostridium* (2% and 2%), *Paenibacillus* (1% and 1%), *Synechococcus* (1% and 1%), and *Sphingobacterium* (1% and 1%) (Figure 4). Also, there were no significant differences between gDNA and cDNA of the predominant ureolytic bacteria.

### 3.4. Changes in the Abundances of OTUs of the UreC Genes

**Figure 5:**
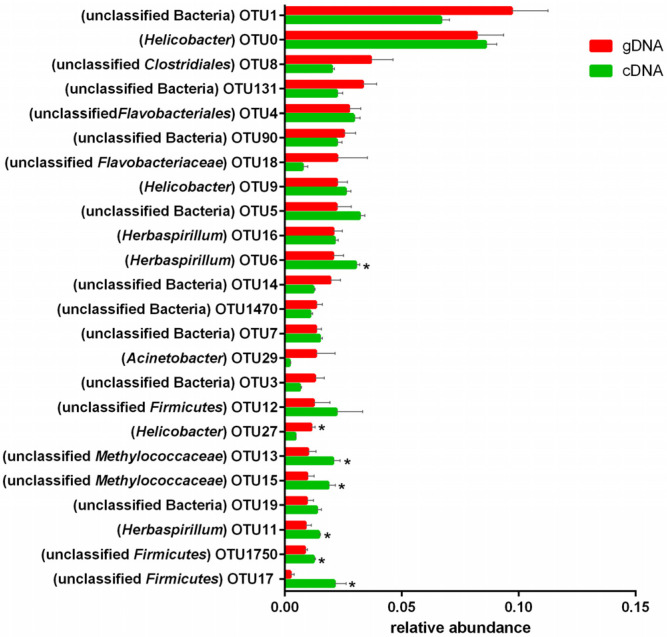
The relative abundance of top 20 *ureC* gene operational taxonomic units. In brackets represent bacteria genus. * *p* < 0.05.

Among the top 20 abundant OTUs of *ureC* genes, 18 (72%) were unclassified ureolytic bacteria at the genus level. The others were mainly distributed in *Helicobacter* (OTU0, 9, 27), *Herbaspirillum* (OTU16, 6, 11), and *Acinetobacter* (OTU29). Among the top 25 OTUs with high abundances of gDNA and cDNA (Figure 5.), there were significant differences in a cluster of OTUs (6, 27, 13, 15, 11, 1750, and 17). Those OTUs with high abundances of gDNA did not necessarily have high abundances of cDNA. Changes to the OTUs revealed that the most abundant *ureC* genes were similar between gDNA and cDNA, and most differences were observed in diverse *ureC* genes with low abundances.

## 4. Discussion

It has been reported that RNA levels are directly related to the synthesis potential and activity of microbial proteins [9,24]. Therefore, the data obtained from cDNA sequencing could potentially be used as an index to taxonomically assess potentially active microbes. Li et al. sequenced targeted RNA and gDNA amplicons to identify and quantify potentially active rumen microbiota, and found significant differences in the taxonomic classifications and community structures [25]. However, sequencing of ureolytic bacteria based on gDNA may be misleading in regard to expressed or active communities. In the present study, quantification of both gDNA and cDNA allowed assessment of the differences in the abundances of major expressed or active and total ureolytic bacteria and the community structure in the rumen. 

In support of this view, the Shannon diversity of cDNA was greater than that of gDNA, sampled at 0 h, indicating a high community diversity of active bacteria. This may be due to the fact that some ureolytic bacteria with lower activity showed transcriptional activity under the stimulation of urea after morning feeding. The Chao1 index of gDNA was greater than that of cDNA after the 2 h feeding, indicating that the total community abundance was higher, which may be due to the basal diet providing adequate amounts of ammonia, amino acids, and/or peptides for the synthesis of microbial proteins [26,27]. However, bacteria can first utilize organic forms of nitrogen for the synthesis of microbial proteins [28]. For samples collected at 6 h after feeding, the Observed_species of cDNA was significantly greater than that based on gDNA, indicating that the community richness of active taxa was much greater, possibly because the amount of protein in the diet was insufficient, which resulted in the bacteria using urea nitrogen for protein synthesis. 

The beta diversity index of the gDNA was significantly greater than that of the cDNA. In the process of urea decomposition, the abundance of active communities was relatively lower, indicating that the expressed or active ureolytic bacteria communities are more concentrated and specific, as not all *ureC* genes coded by gDNA are expressed and actually function. The difference between gDNA and cDNA showed that the active ureolytic bacteria communities were not associated with the total community data [29]. cDNA is probably more indicative of active communities than gDNA, and the dissimilarity in the diversity profiles of each emphasizes that the results from gDNA should be interpreted cautiously with respect to inferences about how communities actively respond to dynamic microenvironments.

Bacteria RNA has a short half-life and is very unstable, the cDNA are active at the time of sampling [30], so the effect was assessed at different time points. The results showed that the sampling time point had no significant impact on the alpha and beta diversity indices of gDNA and cDNA, probably because less ureolytic microbes are active then present. 

The *ureC* gene was used for analysis the ureolytic bacteria. The results showed that the *ureC* gene OTUs were predominately from unclassified taxa. More than 69% and 67% of the *ureC* sequences of gDNA and cDNA, respectively, were not affiliated with any known *ureC* genes at the genus level, indicating that the rumen contained *ureC* genes from unknown sources. Furthermore, most research on urease has been conducted with the use of samples of soils [31,32,33] and ocean water [34,35,36]. Therefore, the reference dataset used for taxonomic assignment of ureolytic bacteria in the rumen need to be updated. 

Changes to the top abundant ureolytic bacteria at the genus level were similar between gDNA and cDNA. The highest proportion of classified *ureC* sequences were from *Helicobacter* sp. Among the predominant OTUs, OTU0 was dominant in cDNA, which was affiliated with *Helicobacter* sp. Hence, a previous study by our group [6] investigated the predominant ureolytic bacteria in the rumen based on gDNA, which revealed a high abundance of *Helicobacter* sp. in the rumen contents. By cloning and sequencing the *ureC* gene, Zhao et al. [37] detected *ureC* diversity in the rumen and found that 22% of the sequences were affiliated with *Helicobacter* sp. Coldham et al. [38] isolated *Helicobacter* sp. from the gastrointestinal tract of sheep that tested positive for urease activity. These findings are consistent with those of the present study, which indicated that *Helicobacter* sp. are the major ureolytic bacteria in the rumen, as determined by the high expression and activity levels. The abundance of *Herbaspirillum* sp. was also relatively high in the rumen, although urease activity was not investigated. *Herbaspirillum* sp. are rhizobacteria that promote plant growth via the ability on fix nitrogen [39]. Diets of cows contain soybeans and silage, which may contain *Herbaspirillum* sp. that are transplanted into the rumen. However, further studies are needed to elucidate the mechanism underlying urea usage in the rumen by *Herbaspirillum* sp. *UreC* genes affiliated with *Clostridium*, *Paenibacillus* and *Synechococcus* sp. were also identified. *Clostridium* sp. reportedly have urease activity [40]. A study conducted by Crociani et al. [41] of urease activity in the stomach (fundus and antrum), content, and soft rabbit feces showed that *Clostridium* sp. also had urease activity. Shi et al. [42] reported that *Paenibacillus polymyxa* strain NSY50 had the ability to increase urease activity by up to 2.25-fold. Jackie et al. [43] cloned and sequenced *Synechococcus* sp. strain WH7805 and found that the WH7805 urease had a predicted subunit composition typical of other bacteria ureases, although the organization of the WH7805 urease genes was unique. The results of these studies are consistent with those of the present study, which detected *ureC* genes of *Sphingobacterium* sp. However, Pinnaka et al. [44] isolated *Sphingobacterium bovisgrunnientis* strain YK2T from yak milk that had no urease activity, likely because the produced urease is active only in the rumen.

This is the first study to compare the ureolytic bacteria composition in the rumen between cDNA and gDNA datasets. The results illustrate differences between active (cDNA) and total (gDNA) ureolytic bacteria communities to describe the composition and characteristics of ureolytic bacteria active in the rumen. A single method cannot fully reveal the composition and diversity of ureolytic bacteria in the rumen. So, the accuracy of the data should be carefully considered in future studies. Difference in total (gDNA) and active (cDNA) ureolytic bacteria communities should be taken into account to better understand the structure and dynamics of ureolytic bacteria in the complex rumen ecosystem. Since the rumen harbors a large diversity of unclassified ureolytic bacteria, future studies are warranted to elucidate the mechanisms controlling urease synthesis.

## 5. Conclusions

In the present study, approximately 69% and 67% of the gDNA and cDNA sequences, respectively, of the ureolytic bacteria community in the rumen of dairy cows could not be confidently classified at the genus level. The sampling time had no significant difference on the alpha and beta diversity indices of gDNA and cDNA. However, the composition of active and total ureolytic bacteria communities were different in the rumen. Moreover, there were no significant differences between gDNA and cDNA profiles of the most abundant ureolytic bacteria, as most differences were observed in *ureC* genes with the lowest abundance. The results contribute new data to ureolytic bacteria information in the cow rumen.

## Supplementary Materials

The following are available online at https://www.mdpi.com/2076-2615/10/11/2162/s1, Figure S1: Alpha diversity of rumen ureolytic bacteria of gDNA and cDNA across different time.

## References

[1] Kertz A. (2010). Review: Urea Feeding to Dairy Cattle: A Historical Perspective and Review. Prof. Anim. Sci..

[2] Owens F.N., Lusby K.S., Mizwicki K., Forero O. (1980). Slow Ammonia Release from Urea: Rumen and Metabolism Studies. J. Anim. Sci..

[3] Jin D., Zhao S., Zheng N., Bu D., Beckers Y., Wang J. (2018). Urea nitrogen induces changes in rumen microbial and host metabolic profiles in dairy cows. Livest. Sci..

[4] Di Jin S.Z., Nan Z., Wang J. (2017). Urea metabolism and regulation by rumen bacterial urease in ruminants—A review. Ann. Anim. Sci..

[5] Alemneh T. (2019). Urea Metabolism and Recycling in Ruminants. Biomed. J. Sci. Tech. Res..

[6] Jin D., Zhao S., Zheng N., Bu D., Beckers Y., Denman S.E., McSweeney C.S., Wang J. (2017). Differences in Ureolytic Bacterial Composition between the Rumen Digesta and Rumen Wall Based on ureC Gene Classification. Front. Microbiol..

[7] De Vrieze J., Pinto A.J., Sloan W.T., Ijaz U.Z. (2018). The active microbial community more accurately reflects the anaerobic digestion process: 16S rRNA (gene) sequencing as a predictive tool. Microbiome.

[8] Villarreal J.V., Jungfer C., Obst U., Schwartz T. (2013). DNase I and Proteinase K eliminate DNA from injured or dead bacteria but not from living bacteria in microbial reference systems and natural drinking water biofilms for subsequent molecular biology analyses. J. Microbiol. Methods.

[9] Blazewicz S.J., Barnard R.L., A Daly R., Firestone M.K. (2013). Evaluating rRNA as an indicator of microbial activity in environmental communities: Limitations and uses. ISME J..

[10] Miller L.L., Ordal Z.J. (1972). Thermal injury and recovery of Bacillus subtilis. Appl. Microbiol..

[11] Rosenthal L.J., Iandolo J.J. (1970). Thermally Induced Intracellular Alteration of Ribosomal Ribonucleic Acid. J. Bacteriol..

[12] Qi M., Wang P., O’Toole N., Barboza P.S., Ungerfeld E.M., Leigh M.B., Selinger L.B., Butler G., Tsang A., McAllister T.A. (2011). Snapshot of the Eukaryotic Gene Expression in Muskoxen Rumen—A Metatranscriptomic Approach. PLoS ONE.

[13] Lanzén A., Jørgensen S.L., Bengtsson M.M., Jonassen I., Øvreås L., Urich T. (2011). Exploring the composition and diversity of microbial communities at the Jan Mayen hydrothermal vent field using RNA and DNA. FEMS Microbiol. Ecol..

[14] MaiväLi Ü., Paier A., Tenson T. (2013). When stable RNA becomes unstable: The degradation of ribosomes in bacteria and beyond. Biol. Chem..

[15] Klein A.M., Bohannan B.J.M., Jaffe D.A., Levin D.A., Green J.L. (2016). Molecular Evidence for Metabolically Active Bacteria in the Atmosphere. Front. Microbiol..

[16] Hugoni M., Agogué H., Taib N., Domaizon I., Moné A., Galand P.E., Mary I. (2015). Temporal Dynamics of Active Prokaryotic Nitrifiers and Archaeal Communities from River to Sea. Microb. Ecol..

[17] Minas K., McEwan N.R., Newbold C.J., Scott K.P. (2011). Optimization of a high-throughput CTAB-based protocol for the extraction of qPCR-grade DNA from rumen fluid, plant and bacterial pure cultures. FEMS Microbiol. Lett..

[18] Bolger A.M., Lohse M., Usadel B. (2014). Trimmomatic: A flexible trimmer for Illumina sequence data. Bioinformatics.

[19] Magoč T., Salzberg S.L. (2011). FLASH: Fast length adjustment of short reads to improve genome assemblies. Bioinformatics.

[20] Caporaso J.G., Kuczynski J., Stombaugh J., Bittinger K., Bushman F.D., Costello E.K., Fierer N., Peña A.G., Goodrich J.K., Gordon J.I. (2010). QIIME allows analysis of high-throughput community sequencing data. Nat. Methods.

[21] Bokulich N.A., Subramanian S., Faith J.J., Gevers D., Gordon J.I., Knight R.T., Mills D.A., Caporaso J.G. (2013). Quality-filtering vastly improves diversity estimates from Illumina amplicon sequencing. Nat. Methods.

[22] Edgar R.C., Haas B.J., Clemente J.C., Quince C., Knight R. (2011). UCHIME improves sensitivity and speed of chimera detection. Bioinformatics.

[23] Edgar R.C. (2010). Search and clustering orders of magnitude faster than BLAST. Bioinformatics.

[24] Dennis P.P., Bremer H. (2008). Modulation of Chemical Composition and Other Parameters of the Cell at Different Exponential Growth Rates. Ecosal. Plus..

[25] Li F., Henderson G., Sun X., Cox F., Janssen P.H., Guan le L. (2016). Taxonomic Assessment of Rumen Microbiota Using Total RNA and Targeted Amplicon Sequencing Approaches. Front. Microbiol..

[26] Agle M., Hristov A.N., Zaman S., Schneider C., Ndegwa P., Vaddella V.K. (2010). The effects of ruminally degraded protein on rumen fermentation and ammonia losses from manure in dairy cows. J. Dairy Sci..

[27] Recktenwald E.B., Ross D.A., Fessenden S.W., Wall C.J., Amburgh M.E.V. (2014). Urea-N recycling in lactating dairy cows fed diets with 2 different levels of dietary crude protein and starch with or without monensin. J. Dairy Sci..

[28] Hristov A.N., Pfeffer E. (2005). Nitrogen and phosphorus nutrition of cattle. Anim. Feed Sci. Technol..

[29] Salazar G., Paoli L., Alberti A., Huerta-Cepas J., Ruscheweyh H.-J., Cuenca M., Field C.M., Coelho L.P., Cruaud C., Engelen S. (2019). Gene Expression Changes and Community Turnover Differentially Shape the Global Ocean Metatranscriptome. Cell.

[30] Gill A.S., Lee A., McGuire K.L. (2017). Phylogenetic and Functional Diversity of Total (DNA) and Expressed (RNA) Bacterial Communities in Urban Green Infrastructure Bioswale Soils. Appl. Environ. Microbiol..

[31] Cordero I., Snell H., Bardgett R.D. (2019). High throughput method for measuring urease activity in soil. Soil Biol. Biochem..

[32] Ashok A., Doriya K., Rao J.V., Qureshi A., Tiwari A.K., Kumar D.S. (2019). Microbes Producing L-Asparaginase free of Glutaminase and Urease isolated from Extreme Locations of Antarctic Soil and Moss. Sci. Rep..

[33] Burbank M.B., Weaver T.J., Williams B.C., Crawford R.L. (2012). Urease Activity of Ureolytic Bacteria Isolated from Six Soils in which Calcite was Precipitated by Indigenous Bacteria. Geomicrobiol. J..

[34] Alonso-Sáez L., Waller A.S., Mende D.R., Bakker K., Farnelid H., Yager P.L., Lovejoy C., Tremblay J.É., Potvin M., Heinrich F. (2012). Role for urea in nitrification by polar marine Archaea. Proc. Natl. Acad. Sci. USA.

[35] Collier J.L., Baker K.M., Bell S.L. (2009). Diversity of urea-degrading microorganisms in open-ocean and estuarine planktonic communities. Environ. Microbiol..

[36] Su J., Jin L., Jiang Q., Sun W., Zhang F., Li Z. (2013). Phylogenetically Diverse ureC Genes and Their Expression Suggest the Urea Utilization by Bacterial Symbionts in Marine Sponge Xestospongia testudinaria. PLoS ONE.

[37] Zhao S., Wang J., Zheng N., Bu D., Sun P., Yu Z. (2015). Reducing microbial ureolytic activity in the rumen by immunization against urease therein. BMC Veter. Res..

[38] Coldham T., Rose K., O’Rourke J., Neilan B.A., Dalton H., Lee A. (2011). Detection, Isolation, and Characterization of Helicobacter Species from the Gastrointestinal Tract of the Brushtail Possum. Appl. Environ. Microbiol..

[39] Marques A.C.Q., Paludo K.S., Dallagassa C.B., Surek M., Pedrosa F.O., Souza E.M., Cruz L.M., Lipuma J.J., Zanata S.M., Rego F.G.M. (2014). Biochemical Characteristics, Adhesion, and Cytotoxicity of Environmental and Clinical Isolates of Herbaspirillum spp.. J. Clin. Microbiol..

[40] Novotný P. (1969). Composition of Cell Walls of Clostridium Sordellii and Clostridium BiferMentans and Its Relation to Taxonomy. J. Med. Microbiol..

[41] Crociani F., Matteuzzi D., Minardi A., Brigidi P., Gioffre F. (1986). Urease activity in gastrointestinal tract of rabbit and electrophoretic behaviour of urease. Ann. Institut. Pasteur. Microbiol..

[42] Shi L., Du N., Shu S., Sun J., Li S., Guo S. (2017). Paenibacillus polymyxa NSY50 suppresses Fusarium wilt in cucumbers by regulating the rhizospheric microbial community. Sci. Rep..

[43] Collier J.L., Brahamsha B., Palenik B. (1999). The marine cyanobacterium Synechococcus sp. WH7805 requires urease (urea amiohydrolase, EC 3.5.1.5) to utilize urea as a nitrogen source: Molecular-genetic and biochemical analysis of the enzyme. Microbiology.

[44] Kaur M., Singh H., Sharma S., Mishra S., Tanuku N.R.S., Pinnaka A.K. (2018). Sphingobacterium bovisgrunnientis sp. nov., isolated from yak milk. Int. J. Syst. Evol. Microbiol..

