# Supplementary material for: Exploring the Diversity of Active Ureolytic Bacteria in the Rumen by Comparison of cDNA and gDNA
Source: Animals (Basel). 2020 Nov 20;10(11):2162. doi: 10.3390/ani10112162 (PMC7699693; doi:10.3390/ani10112162)
Supplement: Supplementary file 1 [file animals-10-02162-s001.pdf]

Supplementary Materials

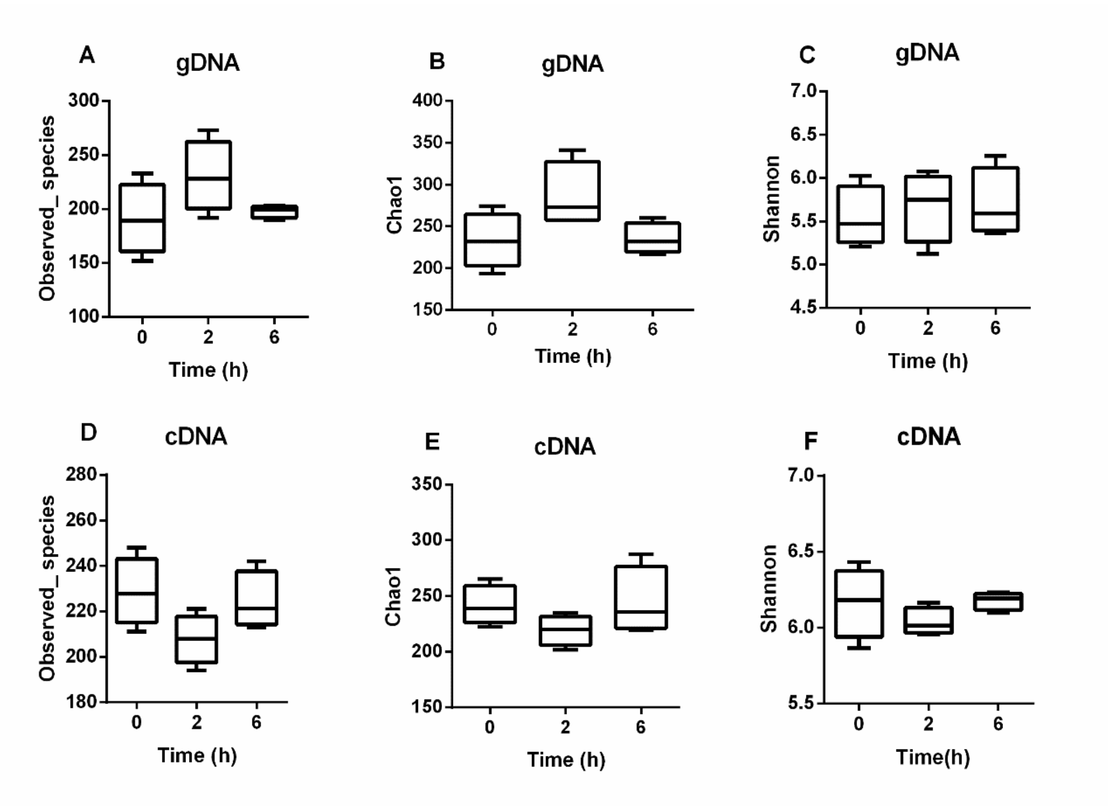

Figure 1. Alpha diversity of rumen ureolytic bacterias of gDNA and cDNA across different time. (**A**) Total observed species (**B**) Chao1 and, (**C**) Shannon index on gDNA. (**D**) Total observed species (E) Chao1 and, (F) Shannon index on cDNA. Boxplots indicate the first and third quartiles with the median value indicated as a horizontal line the whickers extend to 1.5 times the inter quartile range.
